# Essential fatty acids and Barratt impulsivity in gambling disorder

**DOI:** 10.1186/s12888-020-02496-1

**Published:** 2020-03-06

**Authors:** Patricia Sanchez-Paez, Josefa Perez-Templado, Jeronimo Saiz-Ruiz, Oscar Pastor, Angela Ibañez

**Affiliations:** 1 Department of Psychiatry, Hospital Universitario Ramon y Cajal, Madrid, Spain; 2 Universidad de Alcala, Madrid, Spain; 3 Instituto Ramon y Cajal de Investigacion Sanitaria (IRyCIS), Madrid, Spain; 4 Centro de Investigacion Biomedica en Red de SaludMental (CIBERSAM), Madrid, Spain; 5 Department of Biochemistry, Hospital Universitario Ramon y Cajal, Madrid, Spain; 6 Centro de Investigacion Biomedica en Red de Fisiopatologia de la Obesidad y la Nutricion (CIBERObn), Madrid, Spain

**Keywords:** Gambling disorder, Impulsivity, Barratt, Essential fatty acids, Polyunsaturated fatty acids, Omega-3, Omega-6

## Abstract

**Background:**

Polyunsaturated fatty acids (PUFA) have been long implicated in the etiopathogenesis of mental illnesses, including disorders characterized by high impulsivity. The objective of most of the studies in this field is to determine the effect of omega-3 supplementation on the impulsive symptoms. In contrast, studies analyzing basal PUFA composition in patients with impulsive behaviors are very scarce, results are not yet conclusive, and to date, no publication has specifically evaluated this in gambling disorder. Therefore, the main purpose of this research is to examine the relationship between basal PUFA composition of plasma and erythrocyte membrane and impulsivity in subjects with gambling disorder.

**Methods:**

It is an observational and cross-sectional study. The sample consisted of fifty-five men with gambling disorder, who voluntarily accepted to participate. Basal composition of PUFA in plasma and erythrocyte membrane was assessed by gas chromatography and mass spectrometry. Trait impulsivity was measured by the Barratt Impulsiveness Scale version 11 (BIS-11).

**Results:**

Arachidonic acid (AA)/eicosapentaenoic acid (EPA) ratio in the erythrocyte membrane was negatively correlated with total scores in BIS-11. It was also observed that impulsive gamblers had a higher proportion of EPA and a lower value of AA/EPA and AA/docosahexaenoic acid (DHA) ratio in erythrocyte membrane than non-impulsive gamblers.

**Conclusions:**

These results support the hypothesis that alteration of basal PUFA composition exists in disorders characterized by high impulsivity, although the direction of this is still unknown. Unfortunately, the empirical literature on this field is non-existent at the time and we have no direct means to support or refute these outcomes. Further research is needed to determine the relationship between essential fatty acids and disorders characterized by high impulsivity.

## Background

The omega-3 (n-3) and omega-6 (n-6) are essential polyunsaturated fatty acids (PUFA). Humans cannot synthesize them, whereby they must be obtained from the diet or from supplements. Technically, we only need to obtain from dietary resources: alpha-linolenic acid (ALA) of the omega-3 family, to synthesize longer chain n-3, and linoleic acid (LA) of the omega-6 family, to synthesize longer chain n-6. However, studies have shown that humans are inefficient at converting short to long chain fatty acids and thus, the tissue composition of all n-3 and n-6 lipids partially reflects dietary consumption [1]. The main omega-3 derived from ALA are docosahexaenoic acid (DHA) and eicosapentaenoic acid (EPA), which also may be obtained from seafood and plant sources. The main omega-6 derived from LA is arachidonic acid (AA), which depends mainly on the intake of vegetable oils [2].

The role of essential fatty acids (EFA) in brain functioning is currently unknown. However, they are a major structural component of neuronal membranes, and changing the fatty acid composition of neuronal membranes, leads to functional changes in the activity of receptors and other proteins embedded in the membrane phospholipid [3, 4]. They also regulate gene expression involved in neurotransmission and synaptic plasticity [5] and have a role in the inflammatory and cellular signaling through the complex eicosanoid pathway [6].

In addition, it is needed a balance in n-6/n-3 ratio, to maintain an adequate functioning of the organism. However, the value of this ratio is higher than it should be, due to the western diet modification in favor of the increase of omega-6 [7].

The study of essential fatty acids in Psychiatry is interesting because changes in the diet would alter the n-3 and n-6 composition of the neuronal membranes, affecting multiple cellulars and molecular processes involved in the cerebral function and neurotransmission systems associated with pathology. This implies that adequate dietary quantities of PUFA could be a natural, cheap and non-invasive treatment with no adverse effects for the maintenance of mental health [8, 9].

All this biological basis, together with current scientific evidence, supports a possible relationship between EFA and mental disorders, including disorders characterized by high impulsivity, such as attention deficit hyperactivity disorder (ADHD), personality disorders [10], substance use disorders (SUD), suicidal behavior or aggression [11]. Thus, there has been a growing interest in the study of omega-3 in recent years. Most of them have been performed in patients with ADHD, with several meta-analyses recently published. Most have been carried out with the aim of examining the effect of omega-3 supplementation on the symptoms of ADHD [12]. The results obtained in some of them show that omega-3 supplementation could have a small, but beneficial effect on the symptoms of the ADHD, being the EPA the one that seems to reduce more the symptoms [13, 14]. However, there are also some meta-analyses that found no statistically significant benefit [12]. The literature review indicates that small size of the estimated effect of omega-3 supplementation does not provide enough evidence to recommend n-3 as an alternative treatment, although, its use as a supplement along with other treatments is justified because of its favorable side effects profile and the results described. However, despite these promising findings, recent review points to the methodological weaknesses of all clinical trials on which these meta-analyses are based [15]. Issues that might challenge an adequate double-blind placebo-controlled design are various, suggesting to the need for omega-3 clinical trials to adhere to the rigorous standards required of pharmaceutical clinical trials [12].

In contrast, studies analyzing basal PUFA composition in patients with impulsive behaviors are very scarce and results are not yet conclusive. The first meta-analyses that analyzed omega-3 composition in ADHD children find a lower proportion of EPA and DHA in these patients [12]. Controlled trials have also shown an association between suicide attempts and a lower proportion of EPA or DHA in plasma and red blood cells [12]. Likewise, low plasma levels of DHA and high dihomogammalinolenic acid (DGLA) were found in individuals with alcohol abuse and violent behaviors [12]. A recent study finds a negative association between DHA levels and aggression, and a positive association between the ratio AA/DHA and aggression in patients from a forensic psychiatry unit [16].

Nowadays, scientific evidence in animal studies suggests that PUFAs could play a role in the abuse of substances through their action on central serotonergic and dopaminergic systems involved in the reward system [17]. However, studies that directly evaluate the role of EFAs in this type of pathology are scarce and focus on alcohol and cocaine abuse. In the review of this topic by Borsonelo and Galduroz (2008), is concluded the benefit of taking PUFAs as an adjuvant treatment in the alcohol and cocaine use disorders, because of their a role in membrane permeability which could stabilize and repair the damage caused by continued substance use or poor dietary habits of these patients [12]. On the other hand, two studies conducted on patients with cocaine and alcohol dependence reported an association between low levels of omega-3 and high aggressive conduct and this low PUFA status at baseline was a better predictor of relapse than cocaine use, sociodemographic or clinical parameters [12]. These results suggest, but do not prove, the existence of a causal relationship between PUFA status and the vulnerability to the development of addictive disorders, and provide a rationale for the exploration of possible relationships between relapse to addictive disorders and PUFA status in observational and interventional trials.

Few studies have examined a possible relationship between PUFAs and BIS-11 scores, but to date, no publication has specifically evaluated this in gambling disorder [18–20]. The scarce studies in this field, which evaluate a possible effect of fatty acid supplementation on BIS-11 scores, show no enlightening results [21, 22]. In addition, these have numerous methodological limitations.

Reviewing the possible association between basal PUFA composition and scores on the BIS-11, we only found published studies evaluating PUFA in plasma, and none of which evaluates the erythrocyte membrane composition [18–20]. This is striking, considering this method (the composition of PUFA in erythrocyte membrane) is better than the plasma concentration or composition, to provide in clinical practice, a reliable estimate of the basal PUFA values [12], and precisely, this is one of the strengths of the present study.

The main aim of this study is to explore the relationship between PUFA composition of plasma and erythrocyte membrane and impulsivity in subjects with gambling disorder. Our hypothesis is to find a different composition of PUFA among pathological gamblers depending on its impulsivity.

## Methods

### Participants

It is an observational and cross-sectional study. Participants were fifty-five men diagnosed with gambling disorder, who voluntarily attended to the Gambling Addiction Unit of Ramón y Cajal University Hospital to receive counseling and treatment between May 2010 and June 2011.

The inclusion criteria were:

- Caucasian men. The reason for excluded women in the study is because of the high prevalence of gambling disorder among men and the possible differences in both the PUFA and clinical profile of gambling between women and men. So despite being a limitation, it would allow obtaining a more homogeneous sample.
- Over 18 years.
- Diagnoses of pathological gambling per DSM-IV-TR criteria (APA, 2000).

The exclusion criteria were:

- Diagnosis of schizophrenia, bipolar disorder, mental retardation or organic mental disorder.
- Dyslipidemia or current treatment with lipid-lowering drugs.

All subjects give voluntary written informed consent to participate in research under the ethics, consent, and permissions of *Comité de Ética de la Investigación (CEIC) del Hospital Universitario Ramón y Cajal*. The research project was approved by this ethics committee.

Subjects did not receive any incentives for their participation.

### Instruments

#### Medical history

It was collected using a protocolized medical history interview about socio-demographic variables, personal and family history and clinic and history of gambling.

#### Comorbidity

All participants were assessed for comorbidity using the Structured Clinical Interview for DSM-IV-TR Axis I Disorders (SCID I), [23].

#### Dietary habits

Dietary habits were assessed retrospectively by asking about the intake frequency per week of foods rich in EFA (seafood and nuts) during the last year.

*Body mass index (BMI)* and *smoking status* were determined.

#### Diagnostic and statistical manual of mental disorders (DSM-IV)

It is a self-report scale used for the diagnosis of pathological gambling, based on DSM-IV criteria and validated to Spanish. The answer format is yes or no. Scores ranging from 0 to 10 and a value of 5 or more are considered indicative of pathological gambling according to DSM-IV [24, 25].

#### Barratt impulsiveness scale version 11 (BIS-11)

Impulsivity was assessed by this scale. The eleventh revision of BIS-11 [26] is one of the most widely used instruments in impulsivity scientific literature [27]. In this study, we use Spanish validation [28]. It is a thirty question self-report scale that is designed to assess various domains of impulsivity, including attentional impulsivity (ability the sustain attention on a given task), motor impulsivity (ability to control behavioral activity), and non-planning impulsivity (associated with the tendency to think about behaviors and plans before acting). The total score ranges between 30 and 120 and is the result of the sum of three different subscales [29]. It uses a four-point Likert scale from “Rarely/Never” [1] to “Almost Always/Always” [4], where 4 indicates the most impulsive response [12]. A common question asked concerning the BIS-11 is what score can be used to designate an individual as highly impulsive. Several previous studies have used a BIS-11 total score of 74 to designate high impulsiveness [26]. However, current data suggest that a total score of 72 or above should be used to classify an individual as highly impulsive. Thus, Stanford proposes these instrument cut-off points [27]:

- Highly impulsive (higher than or equal to 72).
- Normality (between 52 and 71)
- Low impulsive (lower than or equal to 51).

In the present research, subjects were also categorized into high and low impulsivity, using the same method as Stanford study.

#### PUFA analyses

Fasting blood test was collected under standardized conditions in a 10-mL tube containing EDTA-K3 anticoagulant. Composition of PUFA was analyzed by Biochemistry laboratory by gas chromatography and mass spectrometric detection procedure.

Red blood cells were separated from plasma by low-speed centrifugation. Plasma and packed red cells were stored in cryotubes at − 80 °C until they were analyzed using the direct transesterification procedure according to Lepage and Roy [30]. Samples were analyzed by flame-ionization gas chromatography (HP-INNOWAX®) using a 30 m, 0.25 M, 0.1 mm internal diameter capillary column. One microliter of the sample was auto-injected into the column, and individual fatty acids were quantified. Fatty acid peaks were identified by comparison with known fatty acid standards and quantitated by comparison to the internal standard (GLC-462, NuCheck-INC).

### Statistic analysis

Qualitative data were reported as absolute frequencies and percentages and quantitative data as mean, standard deviation (SD), median, minimum and maximum. Comparison of quantitative variables between groups was performed using the U-Mann Whitney test. Qualitative data were compared using the Chi-square or Fisher’s exact test depending on the distribution. Multiple comparisons between groups were performed by correcting the *p*-value by the Bonferroni method. Correlation between quantitative variables was studied with the Rho Spearman coefficient. All statistical tests were considered bilateral and as significant *p*-values, those less than 0.05. Data were analyzed with the statistical program SPSS 20.0.

## Results

### Sociodemographic characteristics

Sociodemographic characteristics of the sample are described in Table 1.

**Table 1:** Demographic characteristics

Demographic variables	n (%)
Age	
18–29 years	7 (12.7)
30–44 years	25 (45.5)
45–64 years	21 (38.2)
≧65	2 (3.6)
Marital status	
Single	20 (36.4)
Married	25 (45.5)
Divorced or separated	7 (12.7)
Widowed	3 (5.4)
Educational level	
Primary education or less	19 (34.5)
Secondary studies	25 (44.7)
University studies	11 (20.8)
Occupational status	
Student	2 (3.6)
Employed	37 (67.3)
Unemployed	11 (20)
Retired	5 (9.1)

### Clinical characteristics

Clinical characteristics of the sample are shown in Table 2.

**Table 2:** Clinical characteristics

Clinical variables	m (P50)^a^ / n (%)^b^
Type of gambling	
Slot Machines	46 (83.6)^a^
Poker	1 (1.8)^a^
Lottery	4 (7.3)^a^
Casino	4 (7.3)^a^
Last time gambling	
> 60 days	22 (44)^a^
30–60 days	9 (18)^a^
15–30 days	7 (14)^a^
Frequency of gambling	
5–7 days a week	32 (58.2)^a^
3–4 days a week	6 (10.9)^a^
1–2 days a week	9 (16.4)^a^
Hours a day spent gambling	
Hours a day	5.57 (5)^b^

### Comorbidity with substance use disorders

Comorbidity of pathological gambling and substance use disorders (SUD) are shown in Table 3.

**Table 3:** Comorbidity with substance use disorders (SUD)

Comorbidity with substance use disorders (SUD)		
	n	%
Alcohol	21	38.2
Cannabis	14	25.5
Cocaine	12	21.8
Other	3	5.5

### Barratt impulsiveness scale (BIS-11)

The mean of the total score on the BIS-11 was 70.96, with a standard deviation of 10.872 and a median of 71. The minimum value was 50 and the maximum 103. The categorized results from low to high impulsive according to the total scores on the BIS-11 are shown in Table 4.

**Table 4:** Categorization of subjects based on BIS-11

Categorization BIS-11		
	n	%
Low impulsivity (<=51)	1	1.9
Normal impulsivity (52–71)	29	52.8
High impulsivity (> = 72)	24	45.3
Total	54	100

The scores in each of the subscales were:

- Attention impulsivity: mean was 18.5, standard deviation 2.989 and median 18.5. The minimum value was 11 and the maximum 26.
- Motor impulsivity: mean was 22.61, standard deviation 5.101 and median 21. The minimum value was 13 and the maximum 36.
- Non-planning impulsivity: mean was 29.94, standard deviation 5.564 and median 29. The minimum value was 20 and the maximum 46.

The subscales were also categorized into high and low impulsivity, using the same method as in a recent study [31], which established as the cut-off the mean scores of the sample in each of the subscales. They justified not considering the confidence intervals around the mean, corresponding to moderate scores, because of the small sample size.

### Analysis of PUFA and impulsivity

In this study, the possible relationship between essential fatty acids (percent composition in plasma and erythrocyte membrane and plasma concentration) and the impulsivity assessed by BIS-11 were analyzed in the sample of pathological gamblers. There were no statistically significant differences (*p* < 0.05) in age, BMI, fish intake, or smoking among the impulsive and non-impulsive group of pathological gamblers (Table 5). Regarding the possible differences between other sociodemographic variables such as marital status or education, and nut intake, there was not enough sample size to be able to compare groups.

**Table 5:** Age and other variables in subjects with gambling disorder moderated by the Barratt impulsivity scale

Variables m (SD)^a^ /n (%)^b^	BIS-11		Comparison	
	< 72 (*n* = 29)	≧72 (*n* = 24)	Test	*p*-values
Age^a^	41.1 (13.6)	41.3 (9.9)	U Mann Whitney	0.707
BMI^a^	28.3 (5.08)	29.5 (3.7)	U Mann Whitney	0.285
Smoker^b^	18 (62.10)	18 (75)	χ^2^	0.315
Fish^b^	12 (41.4)	12 (52.2)	χ^2^	0.654

It is also recommended to take into account other possible confounding variables like comorbidity with substance use disorders (SUD). Although previous investigations [32] suggest patients with gambling disorder often present a high comorbidity with alcohol or substance abuse/dependence and the most impulsive patients tend to be those with higher dual pathology, in this study no association was found between impulsivity and comorbidity with alcohol or other substance use disorders. However, this could be because our participants were recruited from among treatment seekers and they may differ from the general population of pathological gamblers in certain ways. It is also important to consider that individuals with severe SUD (in which an association with impulsivity could be more easily established), are likely to go to specializing units in such addictive disorders rather than gambling, even if they have a comorbid diagnosis of pathological gambling. In fact, these results are extrapolated to the findings of some previous studies such as Ledgerwood et al. (2009) [33], in which no differences in impulsivity measured by the BIS-11 were found between a group of gamblers with and without comorbidity with SUD.

#### Erythrocyte membrane PUFA composition

As a quantitative variable, lower value of the AA/EPA ratio was associated with higher scores on the BIS-11 (Table 6).

**Table 6:** Correlation between PUFA composition in erythrocyte membrane and total scores on the Barratt impulsivity scale. (Omega 3: EPA, DHA, ALA and Omega 6: AA, LA)

PUFA (% erythrocyte membrane)		Rho de Spearman		
		n	Correlation	*p*-values
BIS-11 Total	Omega 3	50	0.180	0.211
	Omega 6	50	−0.014	0.923
	EPA	50	0.264	0.064
	DHA	50	0.168	0.243
	ALA	50	0.254	0.075
	AA	50	−0.116	0.423
	LA	50	0.224	0.118
	Omega6/Omega3	50	−0.204	0.155
	AA/EPA	50	−0.281	0.048*
	AA/DHA	50	−0.267	0.061

*Abbreviations*: *BIS-11* Barratt Impulsiveness Scale version 11, *PUFA* polyunsaturated fatty acids, *EPA* eicosapentaenoic acid, *DHA* docosahexaenoic acid, *ALA* alpha-linolenic acid, *AA* arachidonic acid, *LA* linoleic acid

*Statistical significance *p* < 0.05

Analyzing the possible association between the composition of PUFA in the red blood cell membrane and scores in the three subscales of BIS-11, the percentage composition of AA/DHA ratio in red blood cells and scores on non-planning impulsivity subscale was found a negative correlation (− 0.283, *p* < 0.05).

It was found statistically significant differences composition of PUFA in the red blood cell between groups. Thus, high impulsive gamblers showed a higher percentage composition of EPA and a lower value of AA/EPA and AA/DHA ratio in erythrocyte membrane than low impulsivity gamblers (Table 7).

**Table 7:** PUFA in subjects with gambling disorder moderated by the Barratt impulsivity scale. (Omega 3: EPA, DHA, ALA and Omega 6: AA, LA)

PUFA (% erythrocyte membrane)	BIS-11 m (SD)		Comparison	
	< 72 *n* = 29	≧72 *n* = 24	Test	*p*-values
Omega 3	4.69 (0.88)	5.42 (1.46)	U Mann Whitney	0.090
Omega 6	28.66 (2.18)	28,.62 (2.72)	U Mann Whitney	0.856
EPA	0.22 (0.09)	0.33 (0.22)	U Mann Whitney	0.022*
DHA	3.37 (0.75)	3.91 (1.09)	U Mann Whitney	0.075
ALA	0.03 (0.01)	0.04 (0.03)	U Mann Whitney	0.056
AA	14.86 (1.82)	14.13 (2.37)	U Mann Whitney	0.284
LA	9.01 (1.01)	10.05 (2.15)	U Mann Whitney	0.051
Omega6 / Omega3	6.35 (1.45)	5.64 (1.61)	U Mann Whitney	0.082
AA/EPA	79.13 (34.75)	55.99 (29.92)	U Mann Whitney	0.015*
AA/DHA	4.74 (1.94)	3.87 (1.24)	U Mann Whitney	0.015*

*Abbreviations*: *BIS-11* Barratt Impulsiveness Scale version 11, *PUFA* polyunsaturated fatty acids, *EPA* eicosapentaenoic acid, *DHA* docosahexaenoic acid, *ALA* alpha-linolenic acid, *AA* arachidonic acid, *LA* linoleic acid

*Statistical significance *p* < 0.05

#### Plasma PUFA composition

It was not found a relationship between the composition of essential fatty acids in plasma and scores on BIS-11 (total and three subscales).

#### Plasma PUFA concentrations

There was no statistically significant correlation between plasma PUFA concentrations and total scores on the BIS-11 (as a quantitative variable). Analyzing each of the subscales, just a weak and negative correlation between ALA (alpha-linolenic acid) and scores on the attentional impulsivity subscale was found (Table 8).

**Table 8:** Correlation between plasma concentrations of PUFA and scores on the Barratt cognitive impulsivity subscale. (Omega 3: EPA, DHA, ALA and Omega 6: AA, LA)

PUFA (plasma concentration)		Rho Spearman		
		n	Correlation	*p*-values
BIS-11 Cognitive Impulsivity	Omega3	52	−0.158	0.264
	Omega6	52	−0.105	0.461
	EPA	52	−0.076	0.594
	DHA	52	−0.172	0.224
	ALA	52	−0.281	0.044*
	AA	52	0.059	0.680
	LA	52	−0.181	0.198
	Omega6/Omega3	52	0.046	0.747
	AA/EPA	52	0.105	0.458
	AA/DHA	52	0.214	0.128

*Abbreviations*: *BIS-11* Barratt Impulsiveness Scale version 11, *PUFA* polyunsaturated fatty acids, *EPA* eicosapentaenoic acid, *DHA* docosahexaenoic acid, *ALA* alpha-linolenic acid, *AA* arachidonic acid, *LA* linoleic acid

*Statistical significance *p* < 0.05

Finally, no statistically significant differences in plasma PUFA concentrations were found between impulsive and non-impulsive gamblers (BIS-11 as a qualitative variable).

## Discussion

The main objective of this study is to analyze the association between plasma PUFA concentration or percentage of PUFA in plasma and erythrocyte membrane, and the impulsivity assessed by the BIS-11 in gambling disorder.

In general, the literature suggests a possible weak and negative correlation between omega-3 levels and the impulsivity assessed by BIS-11 [18–20]. However, it must take into account a variety of methodological limitations (small sample size, cross-sectional studies, lifestyle and diet assessment and concomitant medication intake) [12] and there are also no publications comparing PUFA composition between different categories performed based on total scores on the BIS-11. In summary, publications in this field are very scarce and show no enlightening outcomes. To date, there are no published papers that study PUFA in a sample of pathological gamblers.

The present study agrees partially with previous researches, where no correlation was found between plasma EPA and DHA concentration or composition and BIS-11 scores [18]. Although it should be noted that in Conklin et al. (2007), performed in a non-clinical sample, there was a significant statistically significant weak and negative correlation between plasma concentrations (*r* = − 0.20) and plasma percentage composition (*r* = − 0.26) of alpha-linolenic acid (ALA) and total scores on BIS-11. In another study performed in subjects with depression with and without comorbidity with substances uses disorder and controls, only a negative correlation was observed between plasma EPA (not DHA) and total BIS-11 scores in the group with depression and comorbidity with substance use disorder [19]. Finally, it should be mentioned that in another research that aimed to evaluate the difference between plasma concentration of a control group and a group of subjects with self-harm, there was a statistically significant (*p* < 0.05) weak and negative correlation between plasma omega-3 concentrations (*r* = − 0.23) and omega-6 concentrations (*r* = − 0.3) and the total scores on the BIS-11 [12].

In contrast, this research found a weak and negative correlation (*r* = − 0.281, *p* < 0.05) between the AA/EPA ratio in the red blood cell membrane and the total scores on BIS-11. It was also observed that the impulsivity group showed a higher percentage composition of EPA and a lower value of AA/EPA and AA/DHA ratio in erythrocyte membrane than the non-impulsivity group. These findings support the hypothesis that alteration of basal PUFA composition exists in disorders characterized by high impulsivity, although the direction of this is still unknown. Unfortunately, the empirical literature on this field is non-existent at the time and we have no direct means to support or refute these outcomes.

Analyzing the association between PUFA and the scores on the three subscales of BIS-11, the current research found a weak and negative correlation (*r* = − 0.281, *p* < 0.05), between plasma concentrations of alpha-linolenic acid (ALA) and scores on the attentional impulsivity subscale. This data is interesting and is in line with literature that supports a possible association between decreased levels of omega-3 and higher impulsivity. However, in the Conklin study (2007), this correlation could not be demonstrated, whereas a weak and negative correlation (*r* = − 0.2) was observed between ALA plasma concentrations (*p* < 0.05) and motor impulsivity subscale scores (*r* = − 0.2), and between plasma concentrations of EPA and DHA and scores on the attentional impulsivity subscale (*r* = − 0.2) [18].

To explain these confusing outcomes, we may need to consider that red blood cell PUFA composition could not reflect the central nervous system PUFA composition. Indeed, some studies have found that plasma omega-3 levels decrease was proportional to the decrease ALA intakes, however, the omega-3 brain concentration, strikingly remained stable despite dietary restrictions or supplements [34]. In addition, PUFAs do not have the same capacity to cross the hemato-encephalic barrier and they use different mechanisms to cross it, so it seems wrong to extrapolate the results of what happens in erythrocyte membrane and plasma to the central nervous system [35].

Similarly, it is important to point out that a large volume of the empirical evidence supports an antagonistic action of omega-3 (DHA and EPA) on the signaling of the eicosanoid system dependent on AA metabolism, through several mechanisms [12]. A review of the literature shows that omega-3 and specifically EPA may also have an activating effect on the AA cascade. Although the biochemical mechanisms involved in this process remain unknown, it could be related to the modulation of the activity or expression of the enzymes involved in the release (phospholipase A2) and reuptake (acyltransferase lysophospholipid and CoA fatty acids ligase) of AA in cell membrane phospholipids [12]. This would lead to an increase in AA available for the synthesis of eicosanoids, involved in the modulation and activity of dopaminergic and serotonergic neurotransmission pathways [36, 37], which could eventually lead to the appearance of certain symptomatology.

These mechanisms could explain and give coherence to the finding of higher percentage composition of EPA in the erythrocyte membrane in the impulsive group, due to this higher proportion of EPA could result in damage to brain functioning.

However, it should be mentioned that it is complex to determine whether neurobiological variations precede or follow the development of mental disorders, and in this case, as a cross-sectional study, a causal relationship cannot be established. Although in this sample, no significant correlation between PUFA levels and intake of seafood or nuts has been observed, and previous studies say that plasma levels of omega-3 correlate only modestly with omega-3 intake from dietary assessments [38], we cannot depreciated completely the role of the dietary in these results or the biological variation between individuals (e.g. differences in absorption and metabolism). Also, point out that the outcomes may be affected by the pharmacological treatment of patients.

The main limitations of the present study are the lack of control group and the small sample size. The generalizability of the current findings is limited by the sample size, demographic and clinical features of the selected sample.

Therefore, future researches should include control group and larger and more generalizable samples. In addition, it would be interesting to have other impulsivity measures, including neuropsychological tests and behavioral tasks, since the multidimensionality and different facets of impulsivity may not be well assessed through BIS-11. Moreover, the use of a validated and standardized outcome measures of impulsivity and PUFA should be implemented when conducting research in this field.

## Conclusions

The results obtained support the hypothesis of a different composition of PUFA among pathological gamblers depending on its impulsivity, showing a higher percentage composition of EPA and a lower AA/EPA ratio and AA/DHA ratio in red blood cell membrane impulsive gamblers. However, as suggested by a part of the literature, these results could go contrary to expectations. These unexpected findings indicate a need for additional investigations to understand the role of EFA in brain functioning, which is even more complex than we can know today.

Therefore, available evidence does not permit us to conclude that a causal relationship exists between n-6 or n-3 PUFA status and impulsivity in pathological gambling, but it provides a rationale for further exploration of links between disorders characterized by high impulsivity and basal PUFA, as well as for interventional trials.

Work in this field could lead to the use of treatments that are both well tolerated and inexpensive. In all, it is important to discover whether there is a target subgroup of more impulsive gamblers that would preferentially benefit from a supplementation.

## Data Availability

The datasets used and/or analyzed during the current study are available from the corresponding author on reasonable request.

## Abbreviations

AA: Arachidonic acid
ADHD: Attention deficit hyperactivity disorder
ALA: Alpha-linolenic acid
BIS-11: Barratt Impulsiveness Scale version 11
DHA: Docosahexaenoic acid
DSM-IV: Diagnostic and Statistical Manual of Mental Disorders
EFA: Essential fatty acids
EPA: Eicosapentaenoic acid
LA: Linoleic acid
PUFA: Polyunsaturated fatty acids
